# Simple potentiometry and cyclic voltammetry techniques for sensing Hg^2+^ ions in water using a promising flower-shaped WS_2_-WO_3_/poly-2-aminobenzene-1-thiol nanocomposite thin film electrode

**DOI:** 10.1039/d3ra07932e

**Published:** 2024-01-26

**Authors:** Maha Abdallah Alnuwaiser, Mohamed Rabia

**Affiliations:** a Department of Chemistry, College of Science, Princess Nourah Bint Abdulrahman University PO Box 84428 Riyadh 11671 Saudi Arabia maalnoussier@pnu.edu.sa; b Nanomaterials Science Research Laboratory, Chemistry Department, Faculty of Science, Beni-Suef University Beni-Suef 62514 Egypt mohamedchem@science.bsu.edu.eg

## Abstract

A highly promising flower-shaped WS_2_-WO_3_/poly-2-aminobenzene-1-thiol (P2ABT) nanocomposite was successfully synthesized *via* a reaction involving 2-aminobenzene-1-thiol, Na_2_WO_4_, and K_2_S_2_O_8_ as oxidants. The WS_2_-WO_3_/P2ABT nanocomposite demonstrated remarkable potential as a sensor for detecting harmful Hg^2+^ ions in aqueous solutions. The sensing behavior was evaluated over a wide concentration range, from 10^−6^ to 10^−1^ M, using a simple potentiometric study on a two-electrode cell. The calibration curve yielded an excellent Nernstian slope of 33.0 mV decade^−1^. To further validate the sensing capabilities, cyclic voltammetry was employed, and the results showed an increasing trend in the cyclic voltammetry curve as the Hg^2+^ concentration increased from 10^−6^ to 10^−1^ M with an evaluated sensitivity of 2.4 μA M^−1^. The WS_2_-WO_3_/P2ABT nanocomposite sensor exhibited exceptional selectivity for detecting Hg^2+^ ions, as no significant effects were observed from other interfering ions such as Zn^2+^, Ni^2+^, Ca^2+^, Mg^2+^, Al^3+^, and K^+^ ions in the cyclic voltammetry tests. Furthermore, the sensor was tested on a natural sample that was free of Hg^2+^ ions, and the cyclic voltammetry curves did not produce any characteristic peaks, confirming the sensor's specificity for Hg^2+^ detection. The sensor's cost-effectiveness and ease of fabrication present the potential for developing a simple and practical sensor for detecting highly poisonous ions in aqueous solutions.

## Abstract 2

The WS_2_-WO_3_/P2ABT nanocomposite sensor for Hg^2+^ ions uses both the cyclic voltammetry (three-electrode cell) and the simple potentiometric (two-electrode cell) techniques. The calibration curve yields a Nernstian slope of 33.0 mV decade^−1^ with a sensitivity of 2.4 μA M^−1^.

## Graphical abstract

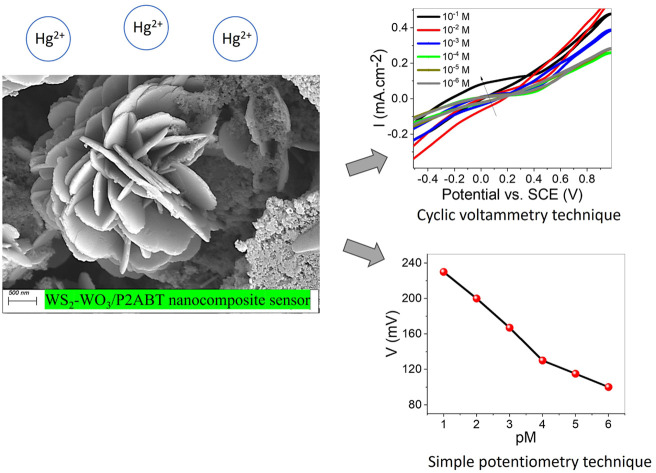

## 1 Introduction

Some vital organic molecules depend on inorganic mineral ions for their vital roles; however, these minerals may show some toxicity and are present in the environment for long periods because they are vital, which makes them common pollutants in the world. As mentioned above, and due to their importance, it is necessary to monitor and determine the levels of heavy metals in the environment, water, soil, and air. Potentiometric electrochemical analysis techniques are indeed simple and promising methods used for various purposes, especially in fields such as analytical chemistry and electrochemistry. These techniques rely on the measurement of potential (voltage) between two electrodes, which can provide valuable information about the analyte of interest. Some common potentiometric techniques include pH measurements, ion-selective electrodes, and redox potential measurements.^1,2^ The permissible limit for most toxic heavy metals according to the Environmental Protection Agency is less than 10 parts per billion (10 ppb) in groundwater, while according to WHO, the limit of Hg^2+^ ions is 1.0 ppm.^3^

In the medical field, identifying mercury or mercury dioxide is important for diagnosing mercury poisoning in patients. Mercury exposure, especially from sources such as contaminated fish or dental amalgams, can lead to severe health issues. Early detection of mercury levels in patients allows for timely intervention and appropriate treatment to mitigate the toxic effects.^4^

In environmental analysis, the detection and quantification of mercury in various matrices such as air, water, soil, and other environmental samples is crucial for evaluating pollution levels and understanding potential ecological risks. Accurate measurement of heavy metals, particularly mercury, is essential for effective environmental monitoring, ensuring compliance with regulatory standards, and developing strategies to mitigate mercury contamination and its potential adverse effects on ecosystems and human health.^5–8^

Some studies based on the detection and quantification of heavy metals in biomedia through color analysis demand a combination of technical expertise, sophisticated equipment, and rigorous protocols to overcome the challenges posed by their low concentrations and potential sample contamination. Nevertheless, these efforts are essential to gain insights into the presence and levels of these metals in biomedia, contributing to a deeper understanding of their role in biological processes and potential health implications. These studies face challenges related to high costs and highly complex techniques. Several sophisticated and intricate analytical methods are employed, including atomic absorption spectroscopy (AAS), mass spectrometers, flame atomic absorption, X-ray fluorescence (XRF), laser thermal lens spectroscopy, neutron activation analysis, plasma block associated with research, and optical emission spectroscopy of plasma. These techniques are characterized by their extensive time requirements and substantial operational costs.^9–12^

An economical technique for detecting mercury or mercury dioxide is highly beneficial as it promotes widespread accessibility and adoption, particularly in settings with limited resources. These cost-effective methods can find applications in various sectors, including medical facilities, environmental monitoring agencies, research institutions, and community initiatives, enabling extensive monitoring, and management of mercury pollution. The use of affordable approaches for mercury identification contributes to the protection of public health, environmental preservation, and sustainable development by effectively addressing the risks associated with mercury exposure and contamination.

Herein, a flower-like WS_2_-WO_3_/P2ABT nanocomposite was effectively synthesized and employed as a sensor for detecting Hg^2+^ ions that showed promising performance. The sensing capabilities of this nanocomposite sensor were evaluated using both two and three-electrode cells, employing simple and cyclic voltammetry systems. Calibration curves obtained from the simple potentiometric study confirmed the excellent sensitivity of the WS_2_-WO_3_/P2ABT sensor to Hg^2+^ ions. The calibration curve demonstrates a clear and linear relationship between the potential response and the concentration of Hg^2+^ ions, showcasing the sensor's ability to accurately detect varying Hg^2+^ concentrations.

Similarly, the cyclic voltammetry curves further validated the sensor's sensitivity to Hg^2+^ ions. The curves exhibited increasing responses as the Hg^2+^ concentration increased, and the area under the cyclic curve that was typically located at 0.1 V increased accordingly. These findings reinforced the nanocomposite sensor's effectiveness in detecting Hg^2+^ ions with high sensitivity. Furthermore, the nanocomposite sensor exhibited remarkable selectivity for Hg^2+^ ions, as observed in the cyclic voltammetry tests. Interfering ions, including other metal ions, are shown to have no effects on the sensor's sensitivity behavior. The absence of characteristic peaks in the cyclic voltammetry curves for interfering ions confirmed the sensor's ability to specifically target and respond to Hg^2+^ ions while avoiding false positive signals from other elements. The combination of simple and cyclic voltammetry techniques, along with the excellent sensitivity and selectivity demonstrated by the WS_2_-WO_3_/P2ABT nanocomposite sensor, highlights its potential for practical applications in Hg^2+^ detection. The sensor's reliability, sensitivity, and selectivity open up possibilities for environmental monitoring, industrial analysis, and health-related applications, contributing to improved safety and environmental protection.

## 2 Experimental section

### 2.1. Materials

Sodium tungstate (Na_2_WO_4_) and sodium arsenite (NaAsO_2_) were sourced from Win lab, UK. HCl and 2-aminobenzene-1-thiol were sourced from Merk (Germany). NaOH and potassium persulfate (K_2_S_2_O_8_) were purchased from Pio Chem company, Egypt. Additionally, dimethylformamide (DMF) was supplied by Merck, USA.

The elemental composition was determined through XPS analyses conducted with equipment from Kratos Analytical in Manchester, UK. Crystalline characteristics were identified using XRD (X'Pert Pro) based in Almelo, The Netherlands. Furthermore, the FTIR analysis was performed using a Bruker device from Easton, USA. In addition, topographical and morphological features were observed using TEM (EOL JEM-2100) and SEM (ZEISS) based in Oberkochen, Germany.

### 2.2. Preparation of WS_2_-WO_3_/P2ABT nanocomposite

The process of oxidative polymerization plays a pivotal role in the creation of P2ABT. In this procedure, 0.06 M of the monomer 2ABT was dissolved in 1.0 M hydrochloric acid (HCl), and an oxidizing agent (0.14 M) was employed to transform the monomer into P2ABT by initiating the formation of free radicals, in which the total volume was 100 ml. This chemical reaction occurred at ambient room temperature and was continued for 24 h. Following the appropriate treatment, the resultant polymer was derived and prepared for utilization in subsequent applications.

The fabrication of the WS_2_-WO_3_/P2ABT nanocomposite thin film involves the oxidation of 2ABT, accomplished by employing a mixture comprising 0.06 M (50 ml) of Na_2_WO_4_ and 0.06 M of K_2_S_2_O_8_. By employing the combination of Na_2_WO_4_ as an oxidizing agent in conjunction with K_2_S_2_O_8_, this process resulted in the integration of WO_3_ and WS_2_ into the polymer matrix, facilitating the formation of the composite. Additionally, the presence of Cl^−^ ions leads to physically drawing the polymer network due to the utilization of HCl as the acid medium. This chemical reaction was allowed to proceed for a duration of 24 h to guarantee the successful creation of the desired nanocomposite thin film.

### 2.3. The potentiometric sensing

The WS_2_-WO_3_/P2ABT nanocomposite was utilized as a potentiometric sensor for detecting Hg^2+^ ions. The negative charge present on the nanocomposite enabled the physical attraction of Hg^2+^ ions from the solution, and the concentration of Hg^2+^ ions affected the calibration curve. For evaluating the sensing behavior, a two-electrode cell was employed with a simple potentiometric technique. The WS_2_-WO_3_/P2ABT nanocomposite served as the primary sensing electrode, and a calomel electrode (Hg/Hg_2_Cl_2_) was used as the reference electrode.

To assess sensitivity and the influence of interfering ions, the cyclic voltammetry technique was employed in a three-electrode cell configuration using CHI608E. The WS_2_-WO_3_/P2ABT nanocomposite served as the working sensing electrode, alongside the calomel electrode, and was accompanied by the counter electrode (which was a graphite electrode, 1.0 cm^2^). Moreover, the performance of the sensing technique was validated by testing natural samples using the above-mentioned methodologies. This approach allowed researchers to assess the sensor's reliability and accuracy under real-world conditions and determine its applicability for environmental monitoring and practical applications. As such, the WS_2_-WO_3_/P2ABT nanocomposite demonstrated promising potential as a sensitive and selective potentiometric sensor for Hg^2+^ ions, with its performance validated through both potentiometric and cyclic voltammetry techniques. The sensor's capability to effectively detect Hg^2+^ ions, along with its assessment in natural samples, made it a valuable tool for environmental monitoring and detection of Hg^2+^ contamination in various settings.

## 3 Results and discussion

### 3.1. Analyses

**Fig. 1:**
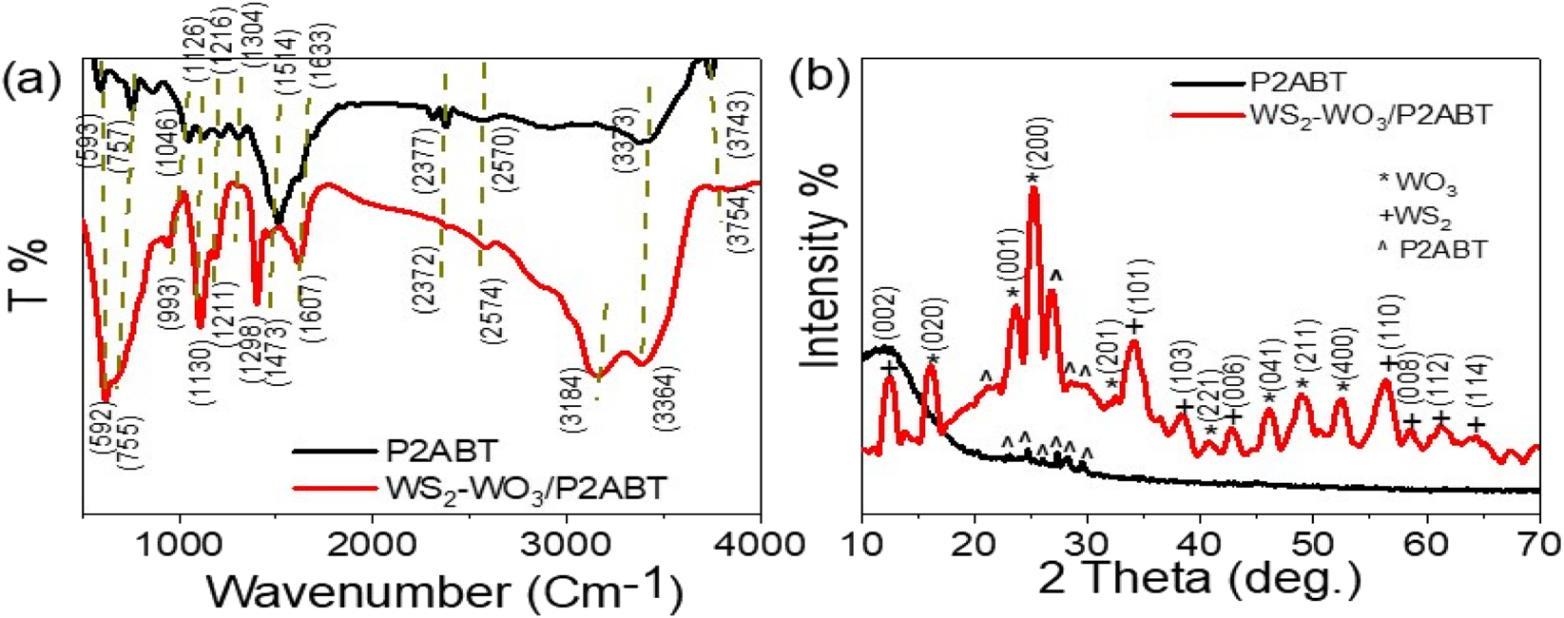
The structural composition of P2ABT and WS_2_-WO_3_/P2ABT nanocomposite through (a) FTIR and (b) XRD analyses.

The structural makeup of P2ABT and the WS_2_-WO_3_/P2ABT nanocomposite was examined by analyzing the positions of functional groups through FTIR analysis, as shown in Fig. 1(a). FTIR analysis allowed us to assess the positions of these functional groups by examining the vibration of bonded electrons. When the polymer matrix was filled with the inorganic material (WS_2_-WO_3_), this filling effect influenced the bond vibrations, leading to observable shifts in the positions of these functional groups in the nanocomposite compared to those in the pure P2ABT polymer. These shifts can be observed as small red or blue shifts, indicating a slight displacement to the right or left, respectively. The summary of the positions of these functional groups can be found in Table 1.

**Table 1:** The band assignments of P2ABT and WS_2_-WO_3_/P2ABT nanocomposites from the FTIR analyses

Band position (cm^−1^)		Function group
WS_2_-WO_3_/P2ABT	POABT	
3754	3743	N–H
3364	3373	S–H^13^
1607	1633	Quinoid C=C
1473	1514	Benzene C=C^14^
1298	1304	C–N^15^
1130	1126	C–H^16^
755	757	*Para* disubstituted ring^17^

XRD patterns of the pure P2ABT polymer and WS_2_-WO_3_/P2ABT nanocomposite are presented in Fig. 1(b). The XRD pattern of pure P2ABT exhibited a semi-crystalline behavior characterized by small, sharp peaks observed in the range of 22.9° to 29.7°. This semi-crystalline nature is advantageous for various applications.

In contrast, the XRD pattern of the WS_2_-WO_3_/P2ABT nanocomposite demonstrated crystalline behavior. It showed a sharp peak at 27° corresponding to P2ABT, along with three smaller peaks at 21.3°, 28.7°, and 30.2°. Additionally,^13^ the pattern revealed highly crystalline peaks for WO_3_ at 16.2°, 23.6°, 25.2°, 41.0°, 46.2°, 49.3°, and 52.6°, corresponding to the growth directions (020), (001), (200), (221), (041), (211), and (400), respectively (based on JCPDS 75-2072 (ref. 18)). Furthermore, the presence of WS_2_ was confirmed by characteristic peaks at 12.6°, 34.3°, 38.55°, 42.7°, 56.5°, 58.7°, 61.4°, and 64.2°, corresponding to the growth directions (002), (101), (103), (006), (110), (008), (112), and (114), respectively.^19,20^

The crystalline size of the prepared WS_2_-WO_3_/P2ABT nanocomposite was determined using the Scherrer eqn (1),^21^ yielding a value of *D* = 23 nm. This evaluation indicates a promising crystalline size for the polymer composites. The calculation is based on the full-width at half maximum value (*β*), which is a measure of the peak broadening, with the taking in consideration of the *λ* = 0.154 nm of the XRD and the angle (*θ*).1
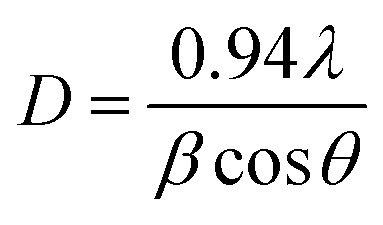


**Fig. 2:**
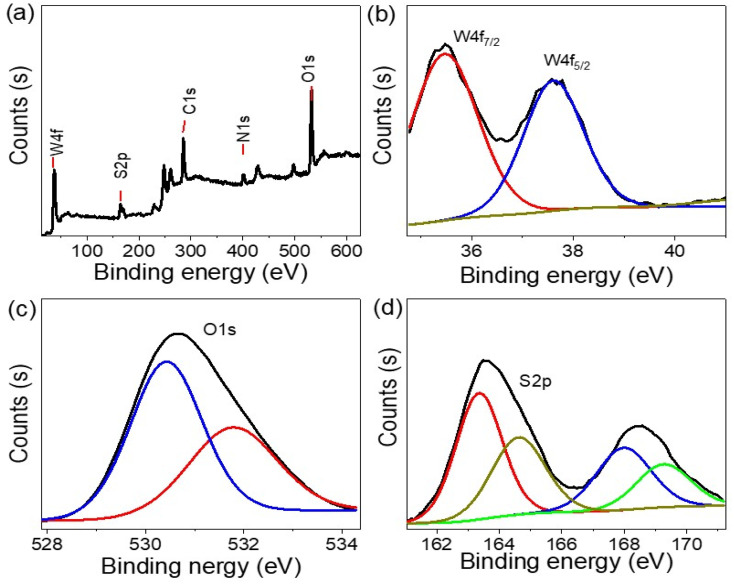
The structural composition of P2ABT and the WS_2_-WO_3_/P2ABT nanocomposite through XPS analyses (a) XPS survey, (b) W 4f, (c) O 1s, and (d) S 2p.

The elemental analysis of the synthesized WS_2_-WO_3_/P2ABT nanocomposite was conducted using XPS, as shown in Fig. 2. The survey scan in Fig. 2(a) confirmed the presence of the elements W, O, S, C, and N in the nanocomposite. The W 4f spectra in Fig. 2(b) revealed the W 4f_7/2_ and W 4f_5/2_ peaks at 35.2 and 37.6 eV, respectively, indicating the formation of WS_2_ (ref. 22) and WO_3_.^23^

The S 2p spectra (Fig. 2(d)), ranging from 163 to 169 eV, corresponds to various sulfur bonds formed with different elements such as S–C and S–W, further confirming the presence of WS_2_ within the polymer matrix. O 1s spectra (Fig. 2(c)) are located at 530.5 and 532 eV,^24,25^ which correspond to O–W bonds. C and N elements were detected at 286 and 402 eV, respectively, consistent with the elements present in the pure P2ABT polymer.

**Fig. 3:**
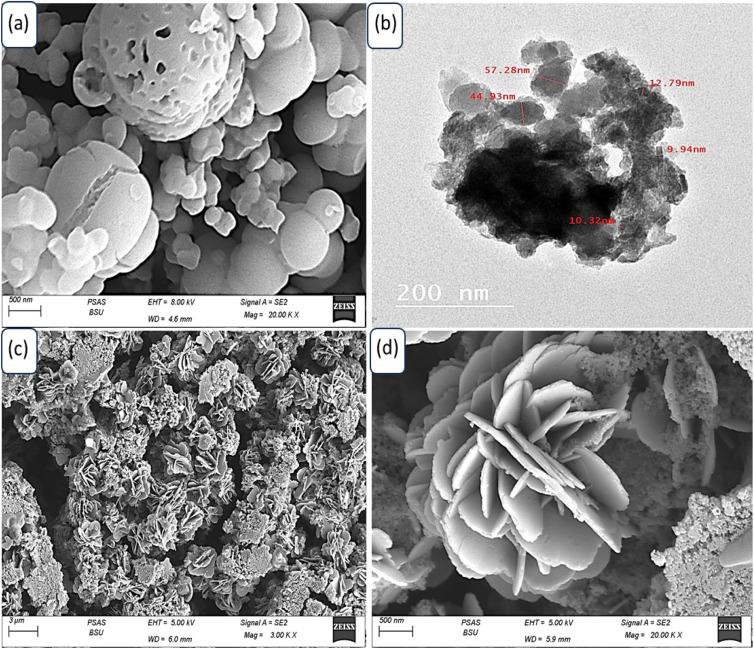
(a) SEM of P2ABT. (b) TEM image and (c and d) SEM images (different scale bar) of the WS_2_-WO_3_/P2ABT nanocomposite.

Fig. 3(a) depicts the morphological characteristics of P2ABT, which exhibits a distinctive cleft spherical ball shape with varying particle sizes ranging from 100 nm to 1 μm. The presence of these larger particles is believed to be the result of a combination of smaller ones during the formation process. The porous structure observed in these particles adds further advantages as it facilitates the formation of composite materials through additional reactions.

Furthermore, the porous structure observed in these particles enhances their surface area and provides additional active sites for potential reactions. This porous characteristic plays a crucial role in facilitating the formation of composite materials when these particles interact with other components in the reaction medium. The increased surface area allows for more extensive interactions and effective incorporation of other materials, leading to the formation of the desired composite structures.

The presence of porous P2ABT particles with a cleft spherical ball shape offers great potential for various applications, especially in composite material synthesis. The unique morphology and porous structure of P2ABT particles open up opportunities for tailored material design and innovative applications in fields such as catalysis, sensing, and energy storage, where composite materials play a significant role.

In contrast, the prepared WS_2_-WO_3_/P2ABT nanocomposite exhibits a novel flower-shaped morphology composed of very small particles with sizes less than 100 nm. These tiny particles aggregate together, giving rise to distinctive and promising flower-shaped structures, as shown in Fig. 3(c and d). The flower-shaped morphology of the nanocomposite is particularly advantageous as it results in leaf-like structures, increasing the material's surface area. This enlarged surface area provides numerous active sites that significantly contribute to the nanocomposite's excellent sensing behavior. The presence of these active sites on the large surface of the flower-shaped nanocomposite enables it to efficiently interact with the target analyte, facilitating highly sensitive and selective sensing. These active sites offer ample opportunities for chemical interactions and binding of the analyte molecules, making the nanocomposite a highly effective sensor.

The flower-shaped morphology and the associated leaf-like structures offer unique features that distinguish the WS_2_-WO_3_/P2ABT nanocomposite from conventional materials. This morphology, along with the abundance of active sites, makes the nanocomposite highly promising for a range of sensing applications, including environmental monitoring, healthcare diagnostics, and industrial process control. The visual representation shown in Fig. 3(c and d) showcases the intricate and fascinating flower-shaped structure of the WS_2_-WO_3_/P2ABT nanocomposite, highlighting its potential for innovative sensing technologies. This remarkable morphology enhances the nanocomposite's performance and makes it a valuable candidate for various sensor applications with heightened sensitivity and selectivity.

The TEM image shown in Fig. 3(b) vividly illustrates the behavior of the WS_2_-WO_3_/P2ABT nanocomposite. In the image, the dark shapes correspond to the inorganic filler, while the gray color represents the polymer materials. The distinct contrast in the TEM image between the dark and gray regions is a clear indication of the segregation of the inorganic filler and the polymer components within the nanocomposite. This segregation is a characteristic feature of the well-dispersed nanocomposites, where the inorganic and polymer phases are spatially separated.

The dark shapes, representing the inorganic filler (WS_2_-WO_3_), are interspersed within the gray polymer matrix (P2ABT). This arrangement indicates a successful synthesis of the nanocomposite, with the inorganic filler being uniformly distributed and embedded within the polymer matrix.

Such a well-dispersed configuration is crucial for optimizing the properties of nanocomposites and performance. The spatial separation of the inorganic and polymer phases ensures that each component retains its unique characteristics, thereby enhancing the overall behavior and functionality of the nanocomposite. The TEM image provides valuable visual evidence of the nanocomposite's structure, reaffirming the successful formation of the WS_2_-WO_3_/P2ABT nanocomposite with a well-defined interface between the inorganic and polymer components. This well-dispersed morphology is pivotal for harnessing the synergistic effects of both materials and realizing the nanocomposite's potential for various applications, including sensing, catalysis, and energy-related technologies.

### 3.2. Sensing properties

**Fig. 4:**
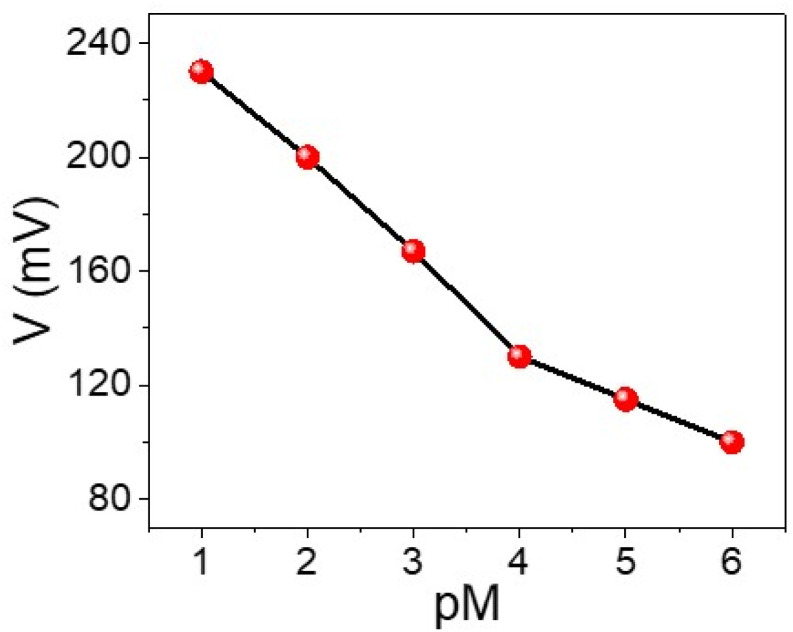
A simple potentiometric method for the detection of Hg^2+^ ions (10^−6^ to 10^−1^ M) using the WS_2_-WO_3_/P2ABT thin film sensor.

The sensing behavior of the WS_2_-WO_3_/P2ABT nanocomposite sensor for Hg^2+^ detection is effectively demonstrated using the simple potentiometric method, as shown in Fig. 4. This sensing method holds great promise for detecting Hg^2+^ ions, as the concentration of Hg^2+^ directly correlates with the potential difference observed between the sensing electrode and the reference electrode.

As the concentration of Hg^2+^ ions increases from 10^−4^ to 10^−1^ M, the potential difference also increases, ranging from 131 mV to 230 mV. The obtained Nernstian slope of 33.0 mV decade^−1^ indicates a highly promising linear relationship between the potential and the logarithm of Hg^2+^ concentration.

The nanocomposite sensor exhibits an impressive detection limit of 9 × 10^−5^ M, showcasing its sensitivity to low concentrations of Hg^2+^ ions. This low detection limit underscores the sensor's capability to detect trace amounts of Hg^2+^ in various sample matrices, which is crucial for environmental monitoring and health-related applications.

Furthermore, the nanocomposite sensor's low-cost and eco-friendly behavior makes it an attractive option for practical applications for Hg^2+^ detection. The use of cost-effective and environmentally friendly materials is advantageous for promoting sustainable and accessible sensing technologies.

Overall, the promising linear behavior, high sensitivity, and cost-effective and eco-friendly nature of the WS_2_-WO_3_/P2ABT nanocomposite sensor make it a highly viable and valuable candidate for Hg^2+^ detection, with potential applications in environmental monitoring and health sciences.

**Fig. 5:**
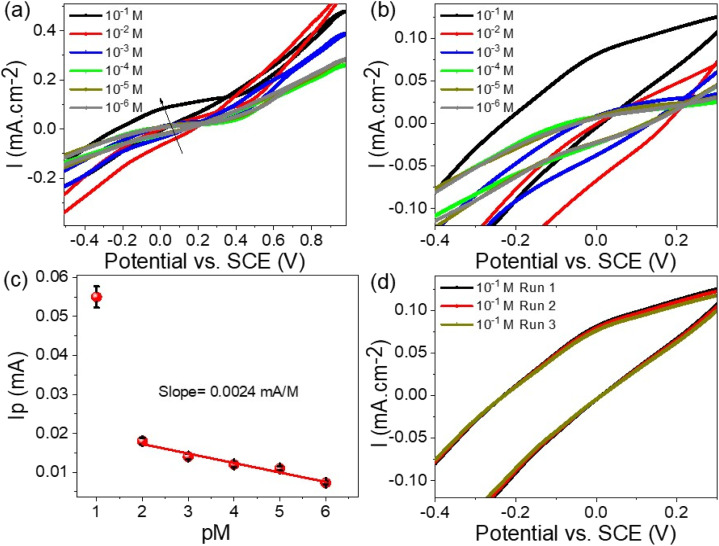
(a and b) WS_2_-WO_3_/P2ABT thin film sensor for Hg^2+^ ions through the cyclic voltammetry, (c) the calculated sensitivity for Hg^2+^ ions, and (d) the reproducibility of the sensor to Hg^2+^ ions.

The detection of Hg^2+^ ions over a concentration range from 10^−6^ to 10^−1^ M was carried out using the WS_2_-WO_3_/P2ABT thin film sensor, employing the cyclic voltammetry technique, as demonstrated in Fig. 5(a). The results showed that the produced current density increases progressively as the Hg^2+^ ion concentration rises from 10^−6^ to 10^−1^ M. This increasing trend is a clear reflection of the high sensitivity of the WS_2_-WO_3_/P2ABT thin film sensor towards Hg^2+^ ions, where higher concentrations lead to a greater potential difference between the sensing and reference electrodes.

As shown in Fig. 5(b), the sensitivity of the WS_2_-WO_3_/P2ABT thin film sensor was quantified, and it was calculated to be 2.4 μA M^−1^. This value indicated how effectively the sensor responded to the changes in the Hg^2+^ ion concentration. A higher sensitivity value suggested that even small variations in Hg^2+^ concentration can be accurately detected by the sensor.

The cyclic voltammetry technique, coupled with the WS_2_-WO_3_/P2ABT thin film sensor, provided a powerful and sensitive means for detecting Hg^2+^ ions across a wide concentration range. The excellent sensitivity and responsiveness of the sensor make it a valuable tool for environmental monitoring and various analytical applications, where precise detection of Hg^2+^ ions is essential.

The combined results presented in Fig. 5(a–c) emphasize the capability of the WS_2_-WO_3_/P2ABT thin film sensor in effectively detecting and quantifying Hg^2+^ ions, making it a promising candidate for practical sensing applications, especially in the fields related to environmental protection and public health. The reproducibility of the WS_2_-WO_3_/P2ABT thin film sensor to Hg^2+^ ions is shown in Fig. 5(d), from this figure, the produced cyclic voltammetry is almost the same value with a very limited standard deviation value. From these data, the limit of detection (LOD) is 0.1 mg L^−1^ which is a promising value for the detection of Hg^2+^ ions.

The accuracy of Hg^2+^ ion recovery can be assessed by examining the current density values obtained from repeated measurements, as illustrated in Fig. 5(c). By conducting the measurements three times and analyzing the resulting current density, it provides a direct reflection of the Hg^2+^ ion concentration. As observed, the recovery values consistently indicated 96.8% across three consecutive measurement cycles. This suggested that the methodology yields a high degree of precision and consistency in estimating the concentration of Hg^2+^ ions, reinforcing the reliability of the analytical approach for Hg^2+^ ion recovery assessment.

**Fig. 6:**
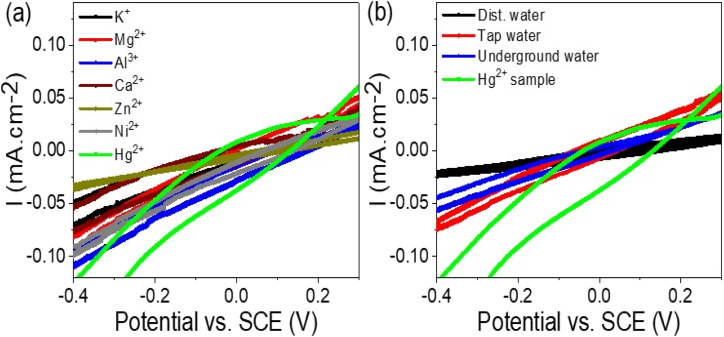
(a) WS_2_-WO_3_/P2ABT thin film sensor for Hg^2+^ ions by testing the response of the (a) interfering ions at 0.01 M and (b) natural samples.

The effectiveness and selectivity of the WS_2_-WO_3_/P2ABT thin film sensor for Hg^2+^ ions were confirmed through the testing of other interfering ions. A series of solutions containing interfering ions, including K^+^, Mg^2+^, Al^3+^, Ca^2+^, Zn^2+^, and Ni^2+^, at a concentration of 0.01 M, were prepared and subjected to the sensor using cyclic voltammetry, as depicted in Fig. 6(a). The results demonstrated that the WS_2_-WO_3_/P2ABT thin film sensor exhibited no response to these interfering elements. No peaks or significant changes in the current density were observed, indicating that these ions do not interfere with the sensing capabilities of the prepared sensor for Hg^2+^ ions.

This lack of response to the interfering ions highlights the sensor's remarkable selectivity for Hg^2+^ ions over other tested elements. The WS_2_-WO_3_/P2ABT thin film sensor exhibited excellent specificity in detecting Hg^2+^ ions even in the presence of potentially interfering ions commonly found in the environmental samples.

The absence of peaks or any significant signal interference from the interfering ions confirmed the high accuracy and reliability of the WS_2_-WO_3_/P2ABT thin film sensor for detecting Hg^2+^ ions. Such selectivity is of paramount importance in practical applications, as it ensures the sensor's capability to precisely identify and quantify Hg^2+^ ions in complex sample matrices.

Overall, the successful validation of the WS_2_-WO_3_/P2ABT thin film sensor's selectivity against interfering ions in Fig. 6(a) further underscores its potential as a robust and accurate tool for Hg^2+^ ion detection in various real-world scenarios, making it an essential asset for environmental monitoring and analytical chemistry applications.

The study of natural samples on the fabricated WS_2_-WO_3_/P2ABT thin film sensor is presented in Fig. 6(b). The natural samples tested included tap water and underground water collected from Egypt, specifically from the Beni-Suef city. Additionally, a laboratory-prepared Hg^2+^ sample, equivalent to the natural samples, was included in the analysis.

The curves obtained from the testing showed that the tap water and underground water samples had no significant effect on the WS_2_-WO_3_/P2ABT thin film sensor. No peaks were observed in the curves, indicating that these natural samples did not interfere with the sensor's performance or generate any false signals.

In contrast, the Hg^2+^ sample exhibited a distinct characteristic peak in the curve, confirming the sensor's sensitivity and selectivity for detecting Hg^2+^ ions. The presence of the characteristic peak in the Hg^2+^ sample curve validated the sensor's ability to accurately identify and quantify Hg^2+^ ions even in complex natural sample matrices. The lack of peaks in the curves of tap water and underground water samples further confirmed the sensor's specificity and reliability in distinguishing Hg^2+^ ions from other components in the natural samples. This level of selectivity is crucial in real-world applications where environmental samples can contain various contaminants and interfering substances.

**Fig. 7:**
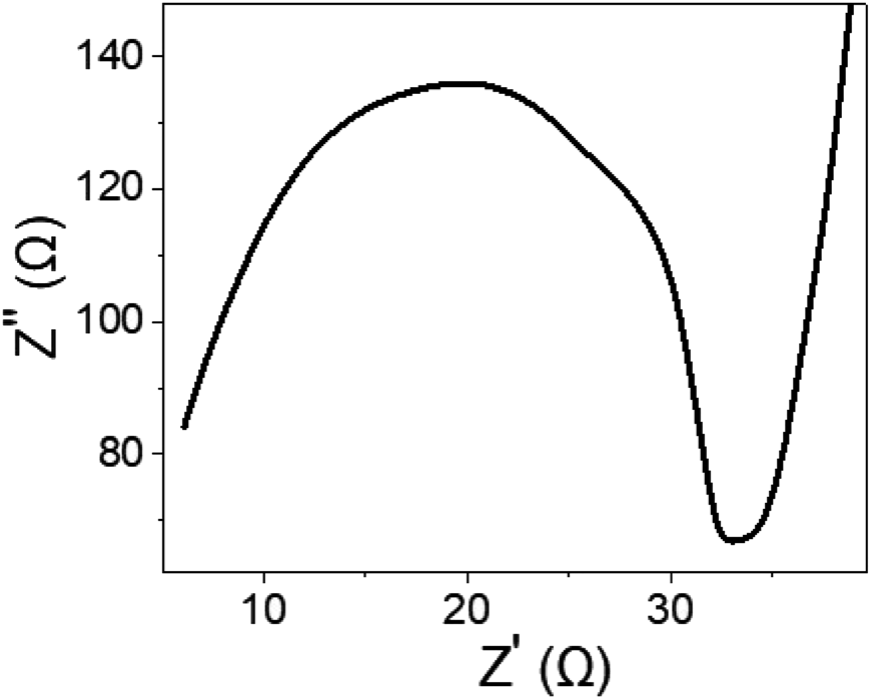
The impedance of the synthesized WS_2_-WO_3_/P2ABT nanocomposite sensor.

To gain further insights into the electrical performance of the fabricated WS_2_-WO_3_/P2ABT nano-composite sensor, impedance measurements were conducted in the presence of 0.01 M Hg^2+^ ions and an additional 0.01 M NaCl electrolyte. The resulting Nyquist plot shown in Fig. 7 reveals a distinct semicircular pattern, indicating a defined electrical response. The solution resistance (*R*_s_) was determined to be 6.0 Ω, while the charge transfer resistance (*R*_CT_) was measured at 27 Ω. These impedance values highlight the promising potential of the sensor for Hg^2+^ ion detection, showcasing its ability to provide a reliable and efficient electrical response. This impedance analysis provided valuable information about the sensor's electrical characteristics, complementing the understanding gained from the *J*_ph_ measurements and emphasizing its suitability for Hg^2+^ estimation.

Overall, the results depicted in Fig. 6(b) demonstrate the excellent performance of the WS_2_-WO_3_/P2ABT thin film sensor for Hg^2+^ detection in natural samples, underscoring its potential as a robust and effective tool for environmental monitoring and water quality assessment. Its ability to accurately detect Hg^2+^ ions amidst diverse natural sample matrices makes it a valuable asset in addressing environmental and health-related concerns associated with mercury contamination.

The rationale behind employing the WS_2_-WO_3_/P2ABT thin film sensor for detecting Hg^2+^ lies in the strong affinity of Hg^2+^ ions for the inorganic WS_2_-WO_3_ materials, forming anticipated partial coordination bonds. Furthermore, P2ABT serves a dual function by establishing physical electrostatic attraction bonds with this metal through both its N and S atoms. The interaction between Hg^2+^ ions and the WS_2_-WO_3_/P2ABT thin film sensor was characterized by charge attraction, resulting in a noticeable shift in the film potential, as evidenced by the produced *J*_ph_ value. In scenarios where the Hg^2+^ concentration is substantial, there is a concurrent increase in the film potential, leading to an elevation in the *J*_ph_ value.^26^ Conversely, as the Hg^2+^ concentration diminishes, the produced *J*_ph_ value exhibits a corresponding decrease. This dynamic relationship underscores the sensitivity of the thin film to Hg^2+^ ions. The charge dynamics within the thin film are instrumental in understanding the sensor's response to varying concentrations of Hg^2+^. The substantial increase in the film potential under higher Hg^2+^ concentrations signifies a heightened affinity between the film and the ions, resulting in an augmented *J*_ph_ value.^21^ This behavior can be attributed to the electrostatic forces between the film and the charged Hg^2+^ ions, influencing the charge carrier density and, consequently, the generated photocurrent.^27^ Additionally, this polymer plays a crucial protective role in safeguarding the sensor against corrosion, thereby enhancing its overall stability. Table 2 illustrates the great behavior of this fabricated sensor related to other previous literature.

**Table 2:** The WS_2_-WO_3_/P2ABT thin film sensor LOD (our study) is compared to other previous studies

Sensor	Linear range (mg L^−1^)	LOD (mg L^−1^)
N-doped WS_2_ (ref. 28)	20–1000	0.4
Al_2_O_3_ (ref. 29)	1–5000	0.35
S–C_3_N_4_–graphene^30^	10.05–1.5075 × 10^3^	0.23
Pt–zeolite^31^	20.1–4.422 × 10^4^	0.68
Graphene–MnO_2_ (ref. 32)	12 000–210 000	2
C-dots^33^	0–1.0	0.21
WS_2_-WO_3_/P2ABT thin film sensor	5 × 10^−3^–0.5	0.1

**Fig. 8:**
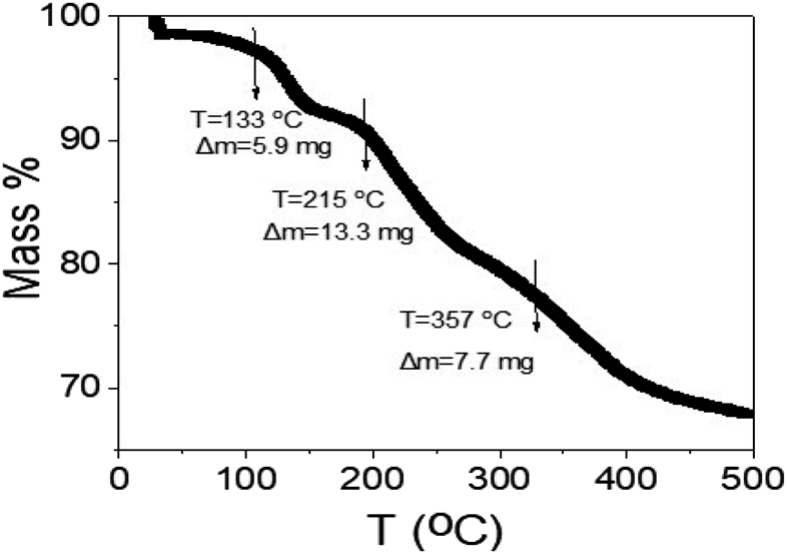
TGA of the synthesized WS_2_-WO_3_/P2ABT nanocomposite sensor.

The thermal gravimetric analyses of the synthesized WS_2_-WO_3_/P2ABT nanocomposite are depicted in Fig. 8. The TGA analysis is conducted in three phases: the initial stage at 133 °C revealed a mass loss of 5.9%, attributed to the removal of the physically adsorbed H_2_O molecules on the nanocomposite particles. The second stage, occurring at 215 °C, corresponds to the partial degradation of the polymer's sulfur component. The final stage, at 357 °C, pertains to the decomposition of the polymer rings and the conversion of WS_2_ into tungsten, accompanied by the degradation of sulfur atoms. This TGA profile exhibits promise for a sensor designed to operate at room temperature, as the cumulative mass loss up to 357 °C reaches 26.9%.

## 4 Conclusions

A promising flower-shaped WS_2_-WO_3_/P2ABT nanocomposite was synthesized from 2-aminobenzene-1-thiol under the effect of Na_2_WO_4_ and K_2_S_2_O_8_, which worked as oxidants. All the characterization behavior for this nanomaterial was performed using XPS, XRD, FTIR, SEM, and TEM. This WS_2_-WO_3_/P2ABT nanocomposite is a promising sensor for the removal of harmful Hg^2+^ ions from aqueous solutions. This testing was performed in a concentration range of 10^−6^ to 10^−1^ M, in which the simple potentiometric study was performed on two electrode cells, and the Nernstian slope of 33.0 mV decade^−1^ was obtained from the calibration curve. This study was confirmed by cyclic voltammetry, in which the cyclic voltammetry curve increases with the concentration from 10^−6^ to 10^−1^ M, with an increasing area under the cyclic curve that is located at 0.1 V, with a sensitivity of 2.4 μA M^−1^.

The WS_2_-WO_3_/P2ABT nanocomposite sensor is selective for the detection of Hg^2+^ ions, while other interfering ions such as Zn^2+^, Ni^2+^, Ca^2+^, Mg^2+^, Al^3+^, and K^+^ ions do not have any effect on the produced current density values of the cyclic voltammetry study. Moreover, this sensor was tested on a natural sample that is free of Hg^2+^ ions, in which the cyclic voltammetry curves did not produce any characteristic peaks. This sensor is cost-effective and easy to fabricate, so it can open the door for a simple sensor for the detection of highly poisonous ions from aqueous solutions.

## Ethical approval

This study does not include any human or animal studies.

## Data availability

All data generated or analysed during this study are included in this article.

## Author contributions

Mohamed Rabia: experimental, analyses, and writing. Maha Abdallah Alnuwaiser: writing, supervision, revision, and ordering the work.

## Conflicts of interest

The authors have no conflict of interest.

## Supplementary Material
